# From Liver to Kidney: The Overlooked Burden of Nonalcoholic Fatty Liver Disease in Chronic Kidney Disease

**DOI:** 10.3390/jcm14072486

**Published:** 2025-04-05

**Authors:** Razvan George Bogdan, Adrian Boicean, Paula Anderco, Cristian Ichim, Mihai Iliescu-Glaja, Samuel Bogdan Todor, Elisa Leonte, Vlad Adam Bloanca, Zorin Petrisor Crainiceanu, Mirela Livia Popa

**Affiliations:** 1 Plastic Surgery Department, “Victor Babes” University of Medicine and Pharmacy, 300041 Timisoara, Romania; razvan.bogdan@umft.ro (R.G.B.); mihai.iliescu.glaja@umft.ro (M.I.-G.); leonte.elisa@umft.ro (E.L.); bloanca.vlad@umft.ro (V.A.B.); crainiceanu.zorin@umft.ro (Z.P.C.); 2 Faculty of Medicine, “Lucian Blaga” University of Sibiu, 550024 Sibiu, Romania; cristian.ichim@ulbsibiu.ro (C.I.); samuelbogdant@gmail.com (S.B.T.); liviamirelapopa@yahoo.com (M.L.P.)

**Keywords:** nonalcoholic fatty liver disease, chronic kidney disease, nonalcoholic steatohepatitis

## Abstract

Nonalcoholic fatty liver disease (NAFLD) is increasingly recognized as a contributor to chronic kidney disease (CKD), yet its impact remains underappreciated in clinical practice. Recent studies reveal a strong association between NAFLD and CKD progression, with evidence linking hepatic dysfunction to renal impairment through metabolic and inflammatory pathways. NAFLD not only increases the risk of CKD but also accelerates its progression, leading to worse cardiovascular outcomes and higher mortality, particularly in patients with advanced fibrosis. Despite this growing evidence, NAFLD often goes undiagnosed in CKD patients and routine hepatic evaluation is rarely integrated into nephrology care. Emerging diagnostic tools, including noninvasive biomarkers and imaging techniques, offer potential for earlier detection, yet their clinical implementation remains inconsistent. Although lifestyle modifications remain the foundation of treatment, pharmacotherapeutic strategies, including SGLT2 inhibitors and GLP-1 receptor agonists, have demonstrated potential in mitigating both hepatic and renal impairment. Recognizing the interplay between NAFLD and CKD is essential for improving patient outcomes. A multidisciplinary approach, integrating hepatology and nephrology expertise, is crucial to refining screening strategies, optimizing treatment, and reducing the long-term burden of these coexisting conditions.

## 1. Introduction

The liver and kidneys share a strong functional relationship, influencing each other in both normal and disease conditions [1]. Renal dysfunction in liver disease can arise from primary conditions such as polycystic liver–kidney disease or as a consequence of hepatitis B and C infections and excessive alcohol consumption [2,3,4]. More recently, nonalcoholic fatty liver disease (NAFLD) has emerged as a notable contributor to the onset of chronic kidney disease (CKD), further highlighting the intricate relationship between hepatic and renal health [5,6]. Both are chronic, progressive disorders that encompass a continuum of pathologies, spanning from mild cases with minimal dysfunction to severe, incapacitating conditions; the disease can progress to end-stage organ failure, ultimately requiring lifelong dialysis or organ transplantation to sustain life [7].

NAFLD encompasses a histopathological spectrum of metabolic liver diseases, including simple hepatic steatosis, characterized by isolated fat accumulation, as well as nonalcoholic steatohepatitis (NASH), which involves steatosis, hepatocellular inflammation, and ballooning, with or without fibrotic progression [8,9]. In its advanced phases, extensive fibrosis or cirrhosis substantially increases the likelihood of hepatocellular carcinoma [9]. NAFLD is the leading chronic liver disease globally, contributing significantly to both hepatic and extrahepatic morbidity and mortality [5]. It affects nearly 30% of the general population in Europe and the USA, with its global prevalence expected to rise further in the coming years [10].

However, the terminology surrounding fatty liver disease has evolved significantly in recent years, prompting revisions to better reflect metabolic contributors [11]. In 2020, a global expert panel introduced the term metabolic dysfunction-associated fatty liver disease (MAFLD), emphasizing a positive diagnostic approach based on evidence of hepatic steatosis along with overweight, type 2 diabetes mellitus, or metabolic dysregulation [11]. More recently, in 2023, a multi-society Delphi consensus proposed the term metabolic dysfunction-associated steatotic liver disease (MASLD) to further standardize the terminology while accommodating coexisting liver conditions [12]. MASLD replaces both NAFLD and MAFLD in clinical and research settings, reinforcing the central role of metabolic dysfunction without excluding individuals based on alcohol intake [12].

Similarly, CKD is a multifaceted, progressive renal disorder, defined by structural or functional kidney abnormalities persisting for over three months, with profound systemic health consequences [13]. This condition is characterized by either biomarkers, indicative of renal damage or a persistent decline in the estimated glomerular filtration rate (eGFR), both of which serve as hallmarks of disease progression [14].

Growing evidence indicates a strong correlation between NAFLD and a higher prevalence and incidence of CKD, characterized by an eRFG below 60 mL/min/1.73 m^2^ (CKD stage ≥ 3) [15]. Chronic kidney disease (CKD) is an established forerunner of terminal renal failure, cardiovascular morbidity and premature death [5,16]. Additionally, NAFLD and CKD are influenced by shared cardiometabolic risk factors and driven by intersecting proinflammatory and profibrotic pathways, further emphasizing their interconnected pathophysiology [7,16].

The strong association between NAFLD and a heightened risk of cardiovascular disease is well-documented across the general population [17,18]. Additionally, emerging research highlights a strong connection between NAFLD and CKD, with CKD prevalence in NAFLD patients ranging from 20% to 55%, compared to 5% to 30% in those without NAFLD [19]. Conversely, 30% to 56% of CKD patients have NAFLD, whereas its prevalence in individuals without CKD is approximately 25% to 30% [19,20,21]. This correlation is expected, given that NAFLD and CKD share multiple risk factors [22,23]. However, the clinical implications of NAFLD in CKD patients remain uncertain [23,24].

The concept of cardiovascular–kidney–metabolic syndrome (CKM syndrome) has recently emerged to describe the interrelated dysfunction of the heart, kidneys, and metabolic systems [25]. This syndrome reflects a multisystem disorder driven by shared risk factors such as obesity, insulin resistance, inflammation, and endothelial dysfunction [25]. Patients with NAFLD and CKD often meet criteria for CKM syndrome, underscoring the need for integrated screening and management. Recognizing CKM syndrome can improve risk stratification and promote multidisciplinary interventions.

## 2. Methodology of Literature Search

This review was conducted following a narrative approach. We searched the PubMed, Uptodate, and Web of Science databases to identify peer-reviewed, English-language articles published up to February 2025. The search strategy combined terms such as “nonalcoholic fatty liver disease” (NAFLD), “metabolic dysfunction-associated steatotic liver disease” (MASLD), “chronic kidney disease” (CKD), “renal impairment”, “hepatorenal axis”, “inflammation”, “hepatokines”, and “cardiometabolic risk”.

We included original research articles, systematic reviews, meta-analyses, and major clinical guidelines or expert consensus statements, prioritizing ISI-indexed publications, prospective cohort studies, and high-quality evidence. The selection was based on clinical relevance, methodological rigor, and their contribution to elucidating the NAFLD–CKD connection. No statistical synthesis was performed due to the heterogeneity of study designs. To ensure comprehensive coverage, additional references were identified by manually screening the bibliographies of key articles.

## 3. Epidemiology

The Centers for Disease Control and Prevention reports that over one-third of U.S. adults are classified as obese, impacting more than 78 million individuals [26]. This disorder plays a major role in the development of insulin resistance, diabetes, and atherosclerosis, imposing an estimated annual healthcare cost of USD 147 billion [26]. The liver, a key regulator of glucose and lipid homeostasis, also serves as a major producer of pro-inflammatory molecules, contributing to the pathogenesis of cardiovascular and kidney disorders [27].

NAFLD is the most widespread chronic liver condition worldwide, impacting nearly a quarter of the global population [28]. Among the various organs impacted by NAFLD, the kidney is a primary counterpart, sharing common metabolic impairments with liver disease [29]. NAFLD is linked to a broad range of kidney impairments, ranging from albuminuria to CKD progression [29]. The connection between NAFLD and CKD is still not fully elucidated and the exact mechanisms linking hepatic and renal dysfunction are still unclear [30]. Similar to type 2 diabetes (T2D), the NAFLD–CKD connection appears bidirectional, with kidney damage further contributing to liver disease progression [31]. A longitudinal study revealed that individuals with T2D and severe NAFLD are at a greater risk of CKD worsening, which serves as a critical predictor of cardiovascular complications and premature death [32]. These results broaden our insight into NAFLD’s impact on diabetic populations in Asia.

Studies suggest that both conditions share key pathogenic mechanisms, including oxidative stress, dysregulation of the renin–angiotensin system (RAS), and gut microbiota alterations [33]. Emerging evidence indicates that NAFLD may actively drive CKD development and progression, rather than merely serving as a marker of kidney disease [7].

A comprehensive meta-analysis encompassing 13 prospective cohort studies and 1.2 individuals in midlife (28.1% diagnosed with NAFLD) established that NAFLD markedly elevates the likelihood of developing stage 3 CKD [5]. This correlation, monitored over a median duration of nearly a decade, persisted regardless of demographic variables, metabolic disturbances, or preexisting renal risk factors, including sex, age, hypertension, obesity, and T2D [5]. NAFLD detection was based on biochemical markers, ICD classification codes, imaging modalities, or histopathological examination, reinforcing its independent role in advancing CKD [5]. A recent umbrella review reaffirmed that NAFLD elevates the likelihood of developing stage 3 CKD, with the association being stronger in women and persisting regardless of obesity status [34]. The study reported a relative risk estimate of 1.42 (95% CI 1.33–1.52) for incident CKD, identical to findings from previous meta-analyses [34]. The risk was strongest in patients with advanced liver fibrosis, but due to the observational nature of the studies, causality cannot be established [34].

CKD is also a widespread public health issue, affecting millions worldwide [35]. Its epidemiology is complex, shaped by factors such as age, sex, ethnicity, and comorbidities [36]. CKD prevalence varies globally, estimated at 15% in the U.S., with higher rates among older adults, African Americans, and Hispanics, while in Asia, it ranges between 10 and 15%, particularly in China and India [36,37]. The occurrence of advanced kidney failure due to CKD also differs across populations, being highest among African Americans in the U.S. and more prevalent in older adults in Japan and South Korea [16,37]. Key risk factors for CKD include diabetes and hypertension, which account for two-thirds of cases globally, along with obesity, smoking, and genetic predisposition [38,39].

## 4. Mechanisms Linking NAFLD to CKD: Hormonal, Inflammatory, and Dietary Pathways

### 4.1. Hepatic–Renal Crosstalk and the Role of Adipose Tissue

The liver–kidney axis in NAFLD and CKD is driven by a combination of genetic, environmental, and metabolic factors [40]. The condition entails a dysfunctional interplay between the liver and kidneys, further exacerbated by adipose tissue, skeletal muscles, and gut microbiota, all of which contribute to disease advancement [41,42]. The pathophysiological mechanisms connecting NAFLD and CKD are not yet fully understood, though several possible explanations have been proposed [43,44,45,46].

RAS is a crucial regulator in the pathogenesis of both NAFLD and CKD. Adipose tissue serves as a major contributor to RAS activity, generating nearly 30% of circulating renin, the angiotensin-converting enzyme (ACE), and angiotensin II, thereby exacerbating metabolic dysfunction and inflammatory signaling pathways [47]. Furthermore, both the liver and kidneys express key components of the RAS, with experimental research highlighting the impact of systemic and localized angiotensin II activation in disease progression [47]. In the liver, angiotensin II contributes to insulin resistance, promotes de novo lipogenesis, and stimulates the secretion of pro-inflammatory cytokines, including interleukin-6 and transforming growth factor-beta, all of which contribute to fibrogenesis and the histopathological changes characteristic of NASH [47,48]. Meanwhile, RAS activation in the kidneys drives abnormal lipid accumulation, fueling oxidative stress and inflammation [49]. This process, facilitated by the vasoconstriction of glomerular efferent arterioles, leads to glomerulosclerosis, aggravating renal dysfunction and accelerating disease progression [49].

Obesity is a standalone risk factor for CKD, linked to proteinuria, podocyte enlargement, and focal segmental glomerulosclerosis, regardless of the presence of diabetes or hypertension [50]. Additionally, both obesity and metabolic syndrome strongly predict NAFLD development [51]. While the complex interplay among adipose tissue, the liver, and the kidneys complicates the precise characterization of the mechanistic pathways connecting NAFLD and CKD, emerging research highlights key pathways such as RAS activation, oxidative stress, and dysregulated lipogenesis [47]. Gaining insight into these mechanisms may facilitate the identification of adjustable risk factors and the development of targeted therapeutic strategies for the prevention and treatment of NAFLD and CKD [48].

**Figure 1:**
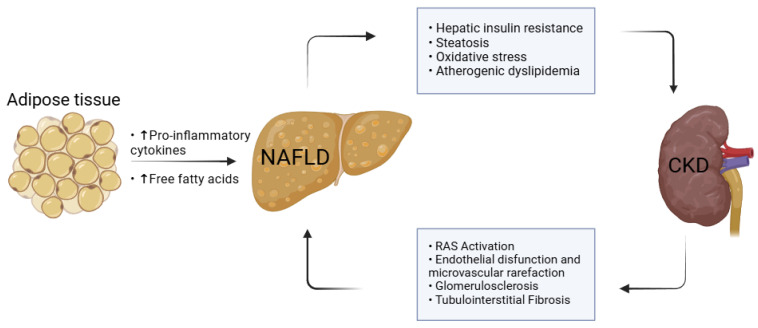
The bidirectional relationship between adipose tissue, NAFLD, and CKD.

Adipose tissue functions as a metabolically active endocrine organ, playing a crucial role in body weight regulation, glucose and lipid metabolism, immunity, and vascular homeostasis [52]. It secretes biologically active adipokines that influence systemic metabolism and growing evidence suggests that these adipokines and inflammatory mediators contribute to the development and progression of both NAFLD and CKD [53,54] (Figure 1). In the setting of NAFLD and CKD, an expanded and inflamed adipose tissue has been implicated in microvascular dysfunction, potentially leading to CKD [7]. Key mechanisms driving this process include impaired insulin signaling, lipotoxicity, oxidative imbalance, RAS activation, the excessive release of proinflammatory cytokines and free fatty acids, as well as decreased concentrations of anti-inflammatory regulators like adiponectin and leptin [41,42]. Elevated circulating leptin levels have been specifically associated with greater NAFLD severity and the progression of CKD, further reinforcing the pathophysiological link between adipose dysfunction and hepatorenal disease [55].

NAFLD and CKD are interconnected through various inflammatory pathways and metabolic disturbances. Elevated levels of pro-inflammatory cytokines such as interleukin-6 (IL-6) and tumor necrosis factor-alpha (TNF-α) are commonly observed in CKD patients, contributing to renal fibrosis and endothelial dysfunction [56]. These cytokines promote tubulointerstitial inflammation by enhancing leukocyte recruitment, activating NF-κB signaling, and stimulating profibrotic factors such as TGF-β, ultimately leading to tubular cell injury and renal fibrosis [57].

Hepatokines, liver-derived proteins like fetuin-A and selenoprotein P, play significant roles in metabolic regulation and inflammation. In NAFLD, the increased secretion of fetuin-A has been linked to insulin resistance and systemic inflammation, potentially exacerbating kidney injury [58]. Similarly, elevated levels of selenoprotein P are associated with impaired glucose metabolism and may contribute to the progression of CKD [59]. Understanding the interplay between these cytokines and hepatokines is crucial for developing targeted therapies to mitigate the progression of both NAFLD and CKD [58,60,61,62].

### 4.2. Free Fatty Acids and Thyroid–Renal Axis

In recent years, growing evidence has highlighted that free fatty acid (FFA) receptors are a newly recognized category of G protein-coupled receptors that regulate site-specific and ligand-activated signaling mechanisms in response to dietary fatty acids [63,64]. Under normal physiological states, FFA receptor signaling is essential for insulin release in response to glucose, the modulation of enterohepatic circulation and enteroendocrine activity, and the regulation of nutrient-dependent energy balance [65]. Additionally, it serves as a critical link between metabolic processes and immune responses by modulating inflammation and peptide hormone secretion [66]. Notably, the discovery of GPR40 and GPR120 in macrophages and neutrophils, respectively, two key regulators of the innate immune system, suggests that FFA receptor signaling could be a promising therapeutic target not only for T2D but also for NAFLD/NASH and related metabolic disorders [64,66].

Thyroid activity plays a fundamental role in maintaining renal circulation, glomerular and tubular efficiency, electrolyte equilibrium, hepatic lipid metabolism, and FFA β-oxidation [67]. Hypothyroidism contributes to hepatic fat accumulation, fostering NAFLD, while hyperthyroidism accelerates its onset by heightening oxidative stress through excessive reactive oxygen species generation [68]. Moreover, the incidence of hypothyroidism escalates with each 10 mL/min/1.73 m^2^ reduction in eGFR, with affected individuals exhibiting a twofold greater likelihood of developing NAFLD and a fourfold higher risk of advancing to NASH [69,70,71]. Table 1 highlights pivotal studies examining the NAFLD–CKD relationship, outlining their methodology, key findings, and clinical implications.

### 4.3. Dietary Pathways and Vitamin D Axis

The excessive intake of sugar-sweetened beverages has been strongly correlated with the emergence of NAFLD, hypertension, metabolic syndrome, and T2D, as demonstrated in both preclinical research and human studies [87,88]. While these links may be partially attributed to excessive caloric consumption and overall lifestyle patterns, high fructose corn syrup remains the predominant source of dietary sugar [7].

Fructose can play a role in hepatic and renal damage through various pathways, including elevated uric acid synthesis [89]. Notably, uric acid-lowering agents have shown potential in improving fructose-induced experimental NAFLD and CKD, highlighting a possible therapeutic approach [33]. Nevertheless, the direct causality between high fructose consumption and metabolic disorders remains a subject of debate and the exact molecular pathways driving fructose-induced liver dysfunction are not yet fully elucidated [87,88].

Higher dietary fructose consumption has been associated with NASH and elevated serum uric acid levels, especially in children and adolescents [90]. Experimental research indicates that fructose metabolism plays a role in NAFLD progression, at least in part, by promoting uric acid production, which triggers mitochondrial oxidative stress and disrupts ATP synthesis [91,92]. Although dietary fructose, mainly from sugar- and corn syrup-sweetened beverages, has been considered the primary source, recent findings suggest that the liver can endogenously produce fructose through the polyol metabolic route [93]. This pathway involves the conversion of glucose to sorbitol by aldose reductase, followed by its transformation into fructose via sorbitol dehydrogenase.

Vitamin D plays a multifaceted role in cell proliferation, differentiation, immunity, inflammation, fibrogenesis, and metabolism [94]. Despite its importance, vitamin D deficiency is highly prevalent, affecting nearly 25% of the adult population [94]. Observational and experimental studies suggest a link between vitamin D deficiency and the pathogenesis and severity of NAFLD and CKD [95,96,97]. However, clinical trials on vitamin D supplementation have produced mixed results, leaving its benefits uncertain [98]. Both NAFLD and CKD are linked to vitamin D resistance, which is partially driven by dysfunctional hepatic 25-hydroxylation and heightened renal tubular excretion of 25-hydroxyvitamin D [99]. Addressing this resistance may necessitate higher-dose supplementation, including calcitriol or vitamin D receptor modulators like paricalcitol [33].

Aldose reductase, an NADPH-dependent enzyme, has recently been implicated in uric acid-induced oxidative stress [100]. Experimental findings indicate that uric acid upregulates aldose reductase expression in a dose-dependent manner, leading to enhanced endogenous fructose synthesis and hepatic triglyceride buildup; this process seems to be driven by the oxidative stress-triggered activation of the nuclear factor of activated T cells 5. Aldose reductase expression is typically low in healthy livers but becomes significantly elevated in fibrotic and cirrhotic livers, particularly within sinusoidal lining cells, Kupffer cells, and fibrotic septa [101]. This upregulation suggests a potential positive feedback loop in NASH, where excess dietary fructose enhances hepatic lipogenesis and uric acid production, leading to oxidative stress [101]. Consequently, oxidative stress may further stimulate aldose reductase expression, perpetuating liver injury and disease progression. The resulting elevation in uric acid levels further stimulates endogenous fructose synthesis via the polyol pathway, exacerbating both hepatic steatosis and kidney damage [100]. This interplay between fructose metabolism, uric acid dysregulation, and oxidative stress highlights a critical mechanistic link between NAFLD progression and CKD risk [7].

## 5. Prevention and Management

Lifestyle changes, such as nutritional modifications and consistent physical exercise, remain the cornerstone of CKD prevention in individuals with NAFLD [65]. In contrast, pharmacological interventions for this population are still in the early stages of development [65].

Noninvasive diagnostic algorithms combining liver fibrosis indices such as FIB-4 with renal markers like the urinary albumin-to-creatinine ratio have shown promise in identifying high-risk patients with both NAFLD and CKD [102]. Emerging radiological tools such as two-dimensional shear wave elastography have demonstrated high diagnostic accuracy for liver fibrosis in NAFLD patients, particularly those with obesity or diabetes [103]. However, the high cost and limited availability of elastography, especially in low-resource settings, pose barriers to widespread implementation [104]. Table 2 summarizes key renoprotective strategies for patients with NAFLD, emphasizing both early screening measures to detect kidney dysfunction and therapeutic interventions aimed at mitigating disease progression and improving long-term renal outcomes.

A healthy lifestyle is typically defined by factors such as moderate alcohol consumption, smoking avoidance, a diet rich in vegetables, limited processed food intake, and regular physical activity. Greater adherence to these lifestyle habits is likely to decrease the risk of CKD in individuals with NAFLD. Reinforcing the strong association between liver histological improvement and lifestyle modifications, a recent study involving 261 patients with biopsy-confirmed NASH demonstrated that a one-stage reduction in liver fibrosis and NASH resolution was linked to enhanced renal function indicators [105]. A research group further investigated this hypothesis in two large-scale prospective cohorts, including the UK Biobank (113,954 participants) and the Chinese Tianzhu County Longitudinal Study on Ischemic Heart Disease (25,974 participants) [106]. By applying a healthy lifestyle score (0–4), which accounted for smoking, alcohol consumption, physical activity, and dietary habits, they determined that adherence to at least three healthy behaviors significantly lowered CKD risk in NAFLD patients over 1,135,334 person-years of follow-up [106].

Glucagon-Like Peptide-1 (GLP-1) receptor agonists offer various therapeutic advantages, such as enhancing insulin release, slowing gastric emptying, and suppressing appetite, which contribute to better blood sugar regulation and weight reduction [107]. Additionally, their anti-inflammatory properties make them promising therapeutic agents for NAFLD and NASH [108]. For example, liraglutide has demonstrated the histological resolution of NASH compared to placebo, though larger trials are needed to confirm these findings [107]. In CKD, GLP-1 receptor agonists exhibit nephroprotective effects, potentially due to their ability to lower blood pressure alongside their metabolic benefits [109].

Both Sodium–Glucose Cotransporter-2 (SGLT2) inhibitors and GLP-1 agonists have well-established cardioprotective properties, which is particularly relevant given the high cardiovascular risk in patients with CKD and NAFLD [110]. Although substantial evidence backs their use in individuals with established cardiovascular disease, data on their preventive role in NAFLD and CKD populations remain scarce. However, in patients with T2D, CKD, and NAFLD, GLP-1 receptor agonists and SGLT2 inhibitors are highly recommended, not only for their glucose-lowering properties but also for their cardioprotective, hepatoprotective, and nephroprotective benefits [111].

Integrating hepatological assessments into nephrology clinics through collaborative care pathways, shared electronic health records, and dual screening protocols has been proposed as a strategy to enhance early diagnosis and optimize the personalized management of patients with coexisting NAFLD and CKD [112]. This multidisciplinary model may improve care coordination, reduce diagnostic delays, and facilitate timely therapeutic interventions [112].

Tirzepatide, a dual GLP-1 and GIP receptor agonist, has demonstrated significant renal protective effects in patients with type 2 diabetes and high cardiovascular risk, including reduced albuminuria, slower decline in eGFR, and a lower incidence of composite kidney outcomes [113].

In patients with T2D, SGLT2 inhibitors have a well-established role in enhancing glycemic control, facilitating weight loss, improving cardiovascular outcomes, and reducing serum uric acid levels [114]. The CREDENCE trial demonstrated that canagliflozin, an SGLT2 inhibitor, significantly improved CKD-related outcomes in diabetic patients [115]. Emerging research indicates that SGLT2 inhibitors may also decelerate NAFLD progression, as evidenced by improvements in transient elastography and reductions in liver enzyme levels [116]. Additionally, their uric acid-lowering effects may further contribute to their benefits in both CKD and NAFLD [117]. Beyond inducing glucosuria, these inhibitors are thought to suppress inflammation and oxidative stress, which are key contributors to the development of NAFLD and NASH [118].

Integrating hepatological assessments into nephrology clinics through collaborative care pathways, shared electronic records, and dual screening protocols could optimize the early detection and personalized treatment of NAFLD–CKD patients [112].

## 6. Discussions

The connection between NAFLD and CKD has drawn growing interest in recent years. NAFLD is recognized as a multifactorial disease, with the multiple parallel hit hypothesis suggesting that hepatic insulin resistance, fat accumulation, inflammation, and chronic oxidative stress drive its progression to NASH [119]. These same mechanisms are also implicated in CKD development and progression, where metabolic dysfunction and systemic inflammation contribute to tubulointerstitial hypoxia, a key driver of CKD progression [120,121].

The molecular mechanisms bridging NAFLD and CKD likely stem from the systemic dissemination of pathological mediators originating from the fat-laden and inflamed liver. These include reactive oxygen species, plasminogen activator inhibitor-1, interleukin-6, C-reactive protein, TNF-α, TGF-β, and a range of pro-inflammatory cytokines, all of which contribute to renal dysfunction and disease progression [15]. Several case–control studies have reported elevated levels of pro-coagulant, inflammatory, and oxidative stress markers in NAFLD/NASH patients compared to those without these conditions [119,122,123,124].

From a pathophysiological viewpoint, persistent inflammation, excessive oxidative stress, and a hypercoagulable state are progressively being identified as critical factors in CKD advancement, as demonstrated in experimental animal models [125,126,127,128]. In CKD, the synthesis and breakdown of both pro-inflammatory and anti-inflammatory cytokines become dysregulated, resulting in an imbalance of immune mediators. This disruption may play a role in renal deterioration through multiple pathways, including the stimulation of inflammatory cascades, increased expression of cell adhesion proteins, vascular endothelial impairment, heightened oxidative damage, and diminished levels of adiponectin [125,126,127,128]. While the exact mechanisms remain unclear, preliminary findings suggest that these detrimental pleiotropic effects play a significant role in CKD pathogenesis and its associated complications.

The connection between NAFLD and CKD has been thoroughly investigated across diverse populations and research methodologies, consistently confirming NAFLD as an independent determinant of CKD progression. A 2014 meta-analysis encompassing 33 studies and 63,902 individuals identified a robust correlation between NAFLD and CKD, revealing that NAFLD nearly doubled CKD prevalence and heightened the risk of CKD onset by 79% [129]. Notably, the severity of NAFLD was correlated with CKD progression, as NASH and advanced fibrosis significantly elevated CKD risk compared to simple steatosis or non-advanced fibrosis [129]. These findings remained independent of diabetes status and other traditional risk factors, highlighting NAFLD as a key driver of CKD development [129].

Building on this, a large database study from Germany provided further evidence of this link, demonstrating that NAFLD patients had a significantly higher incidence of CKD over a 10-year period compared to those without NAFLD (17.1% vs. 11.6%) [130]. This association remained consistent across various age groups and high-risk populations, including individuals with type 2 diabetes and hypertension. These results emphasize the importance of regular kidney function monitoring in NAFLD patients to allow early intervention and slow disease progression [130].

Beyond general populations, a study investigating the NAFLD–CKD relationship in the U.S. and China found that NAFLD was a distinct predictor of risk for CKD in China but not in the U.S. [21]. However, when late-stage CKD cases were excluded, NAFLD was consistently linked to early kidney dysfunction in both populations. These findings suggest that regional differences in CKD stage distribution and dietary habits may explain previous inconsistencies, reinforcing NAFLD as a significant risk factor for early kidney injury [21].

Further research has also explored specific populations and early markers of kidney damage. One study examined NAFLD prevalence in individuals with prediabetes, visceral obesity, and preserved kidney function, focusing on its link to renal hyperfiltration, an early indicator of kidney dysfunction. This suggests that NAFLD may contribute to CKD onset even before significant renal impairment occurs, highlighting the importance of early screening and risk stratification [131].

In addition to adult populations, the impact of NAFLD on renal health has been increasingly recognized in children. A key study on MAFLD and CKD in pediatric patients emphasized the impact of insulin resistance, chronic inflammation, oxidative imbalance, and gut microbiota dysbiosis in the development of hepatorenal dysfunction [132]. Additionally, genetic variants such as PNPLA3, TM6SF2, and MBOAT7 were identified as contributing to both liver and kidney disease, increasing cardiometabolic risk. Given these findings, this study advocates for early screening and a multidisciplinary approach to managing pediatric MAFLD to prevent long-term renal complications [132].

Finally, a study assessing NAFLD risk in younger individuals and those with severe liver disease demonstrated that NAFLD significantly increases CKD risk, independent of traditional cardio-renal risk factors [133]. This highlights the need for targeted risk assessment and early intervention strategies to mitigate CKD progression in high-risk NAFLD patients [133].

## 7. Conclusions

Recent evidence highlights NAFLD as a significant yet often overlooked contributor to CKD, driven by common pathophysiological pathways such as insulin resistance, chronic inflammation, oxidative stress, and lipid dysregulation. The complex interaction between hepatic and renal dysfunction is further influenced by metabolic risk factors, gut microbiota imbalances, and genetic predisposition, accelerating disease progression and systemic complications.

Despite its strong association with CKD progression, cardiovascular disease, and mortality, NAFLD remains underdiagnosed and undertreated in nephrology care. Noninvasive biomarkers, imaging techniques, and risk prediction models offer promising tools for early identification, but their integration into routine CKD management remains limited.

Therapeutic strategies targeting lifestyle modifications (diet, exercise), SGLT2 inhibitors, and GLP-1 receptor agonists have shown promising hepatorenal benefits, yet pharmacological options remain in the early stages of development. Given the bidirectional relationship between NAFLD and CKD, a multidisciplinary approach involving hepatologists, nephrologists, and metabolic specialists is crucial to improving patient outcomes.

Greater awareness, early screening, and proactive management are essential to mitigate the burden of NAFLD in CKD, preventing further disease progression and reducing associated morbidity and mortality. Future studies should prioritize improving diagnostic frameworks, developing precision therapies, and assessing long-term prognoses to strengthen hepatorenal protection in this vulnerable population.

## Figures and Tables

**Table 1:** The link between NAFLD and CKD: key findings from recent studies.

Study	Study Type and Population	Findings	Comments
Park et al., 2019 [45]	Propensity-matched cohort study using a large U.S. insurance database (262,619 NAFLD vs. 769,878 non-NAFLD patients).	NAFLD was associated with a 41% increased risk of advanced CKD, independent of diabetes, hypertension, obesity, and cirrhosis.	This study provides strong epidemiological evidence that NAFLD independently increases CKD risk, suggesting the need for routine renal function screening.
Nah et al., 2022 [72]	Cross-sectional study using data from 13 health-promotion centers in Korea (8909 participants).	NAFLD prevalence was 47.6%, with 12.4% diagnosed with CKD. NAFLD was linked to early CKD but not advanced CKD after adjusting for metabolic factors.	Highlights the impact of NAFLD on early CKD but suggests that metabolic factors play a greater role in advanced CKD.
Mikolasevic et al., 2013 [46]	Fibroscan-based study on 62 CKD patients in Croatia assessing NAFLD prevalence and severity.	85.5% of CKD patients had NAFLD. Liver steatosis severity correlated with serum creatinine and inversely with eGFR.	Demonstrates a strong NAFLD–CKD link using Fibroscan, supporting noninvasive liver assessment in CKD patients.
Tanaka et al., 2023 [73]	Prospective study on 13,159 Japanese adults followed for 10 years.	MAFLD was found to predict new-onset CKD better than NAFLD or simple fatty liver (FL).	This study highlights MAFLD as a stronger predictor of CKD than traditional NAFLD definitions, suggesting that metabolic dysfunction plays a crucial role.
Lau et al., 2010 [74]	Longitudinal study on 3191 individuals from Germany.	FL disease was significantly associated with hypertension and increased blood pressure over time.	Suggests a close link between liver fat accumulation and hypertension, supporting early intervention strategies.
Machado et al., 2011 [75]	Cross-sectional study on 148 morbidly obese patients from Lisbon, Portugal.	NASH was associated with lower eGFR, indicating renal impairment. Advanced fibrosis correlated with greater CKD risk.	Provides strong evidence for a direct liver–kidney connection, particularly in severe obesity and advanced liver disease.
Targher et al., 2008 [76]	Prospective study on 1760 type 2 diabetes patients for over 6.5 years.	NAFLD increased the risk of CKD by 49% even after adjusting for metabolic risk factors.	Reinforces the independent role of NAFLD in CKD development among diabetics.
Yilmaz et al., 2010 [77]	Study on 87 biopsy-confirmed NAFLD patients from Turkey.	Microalbuminuria was more prevalent in patients with higher liver fibrosis scores, linking NAFLD severity to kidney dysfunction.	Indicates that early renal damage in NAFLD may be identified through microalbuminuria, suggesting potential screening markers.
Söderberg et al., 2010 [78]	28-year cohort study on 256 Swedish patients with elevated liver enzymes, focusing on NAFLD-related mortality.	NAFLD increased overall mortality by 69%, with NASH patients facing the highest risk. Liver disease was the third most common cause of death.	Highlights the long-term mortality impact of NAFLD, particularly NASH, reinforcing the need for monitoring.
Campos et al., 2008 [79]	Study on metabolic and inflammatory markers in patients with NAFLD and their potential link to CKD.	Metabolic dysfunction and inflammation in NAFLD patients were associated with kidney function decline.	Supports the role of metabolic and inflammatory pathways in linking NAFLD to CKD progression.
Triozzi et al., 2021 [80]	Retrospective cohort study on 1,155,901 CKD patients without NAFLD at baseline, followed from 2005 to 2016.	NAFLD incidence was higher in earlier CKD stages, associated with metabolic factors like BMI and diabetes. The use of ACE inhibitors was linked to a reduced likelihood of NAFLD, whereas statin therapy appeared to increase the risk.	Emphasizes the metabolic drivers of NAFLD in CKD and highlights ACE inhibitors’ potential protective role.
Adrian et al., 2022 [81]	Cross-sectional study evaluating hepatic liver fat content via CT in 291 CKD patients vs. 866 controls.	No significant association between CKD stage and moderate–severe hepatic steatosis. Diabetes and obesity were major risk factors.	Suggests CKD does not inherently increase NAFLD risk but shares common metabolic pathways.
Choe et al., 2020 [82]	Retrospective cross-sectional study on 819 CKD patients assessing NAFLD risk using noninvasive serum markers.	NAFLD was observed in 15.7% of CKD patients in the derivation group and 20.2% in the validation group. A predictive model using BMI, renal function, triglyceride-glucose index, serum ALT, and hemoglobin achieved an AUROC of 0.850.	This study provides a validated model for predicting NAFLD in CKD patients, emphasizing its comparable prevalence to the general population.
Hydes et al., 2023 [24]	Prospective cohort study using the UK Biobank on 18,073 CKD patients, evaluating NAFLD impact on adverse outcomes.	NAFLD was associated with increased risks of cardiovascular events (HR 1.20) and all-cause mortality (HR 1.31). Advanced liver fibrosis further elevated risks.	This large-scale study highlights NAFLD’s role in worsening CKD outcomes, reinforcing the need for early screening and intervention.
Roderburg et al., 2023 [83]	Retrospective study using the IQVIA database, analyzing 92,225 NAFLD and matched non-NAFLD patients over 10 years.	CKD incidence was significantly higher in NAFLD patients (19.1% vs. 11.1%). The risk was more pronounced in younger (18–50 years) and female patients.	This large real-world cohort confirms NAFLD as a strong independent risk factor for CKD, advocating for interdisciplinary care.
Jang et al., 2018 [84]	Cohort study of 1525 CKD patients investigating NAFLD’s impact on renal function decline.	NAFLD was linked to a faster decline in eGFR, with severity correlating with greater deterioration. Smoking and hypertension exacerbated this progression.	Provides strong evidence for NAFLD’s role in CKD progression, advocating for targeted interventions in high-risk groups.
Takahashi et al., 2021 [85]	10-year longitudinal study on 14,163 healthy individuals assessing FL index as a predictor of CKD.	Higher FLI levels independently predicted CKD development, with the risk increasing across FLI tertiles.	Demonstrates the utility of FLI as a noninvasive predictor for CKD, emphasizing its clinical relevance in early risk assessment.
Kasem et al., 2023 [86]	Cross-sectional study on 430 Egyptian patients evaluating NAFLD and CKD associations.	CKD prevalence was significantly higher in NAFLD patients (38.1% vs. 7.4%), with hypertension and diabetes being major risk factors.	Confirms the bidirectional risk between NAFLD and CKD, underlining the need for integrated disease management strategies.

**Table 2:** Renoprotective strategies in NAFLD: screening, monitoring, and treatment approaches.

Category	Recommended Measures	Description
Screening and Monitoring	Serum Creatinine and Cystatin C	Assess kidney function and detect early renal impairment
	eGFR	Evaluate kidney filtration capacity
	Urine Albumin-to-Creatinine Ratio and Protein Excretion	Detect early kidney damage and albuminuria
	Imaging: Renal Ultrasound and/or Computed Tomography	Assess structural kidney changes
	Blood Pressure Monitoring	Identify and manage hypertension, a key CKD risk factor
Therapeutic Strategies	Lifestyle Modifications	Mediterranean diet, weight management, and regular physical activity
	Optimizing Glycemic Control	Maintain blood glucose levels within target ranges to reduce renal stress
	Blood Pressure and Lipid Management	Control hypertension and dyslipidemia to slow CKD progression
	Pharmacologic Interventions	Use of evidence-based agents such as SGLT2 inhibitors, RAAS blockers, and GLP-1 receptor agonists
	Reducing Uric Acid Levels	Consider xanthine oxidase inhibitors in hyperuricemic patients
	Managing Gut Dysbiosis	Probiotics and dietary fiber to improve gut–liver–kidney axis

## Data Availability

Not applicable.
